# Protocol for a systematic review of good surgical practice guidelines for experimental rodent surgery

**DOI:** 10.1136/bmjos-2022-100280

**Published:** 2022-09-05

**Authors:** Felix Gantenbein, Tim Buchholz, Kimberley Elaine Wever, Merel Ritskes Hoitinga, Stephan Zeiter, Petra Seebeck

**Affiliations:** 1 Zurich Integrative Rodent Physiology (ZIRP), University of Zurich, Zurich, Switzerland; 2 AO Research Institute Davos, Davos, Switzerland; 3 Institute for Health Sciences, Department of Anesthesiology, Radboud University Medical Center, Nijmegen, The Netherlands; 4 Utrecht University, Utrecht, The Netherlands; 5 AUGUST, Department for Clinical Medicine, Aarhus University, Aarhus, Denmark

**Keywords:** biomedical research, mice, models, animal, rats, standard of care

## Abstract

**Objective:**

Surgery is an integral part of many experimental studies. Aseptic and minimal invasive surgical technique and optimal perioperative and post-operative care are prerequisites to achieve surgical success and best possible animal welfare outcomes. Good surgical practice cannot only improve the animal’s postoperative recovery, but also study outcome and validity. There seems to be a lack of implementation of good surgical practice during rodent surgery. The aim of this systematic review is to identify, critically evaluate and compare the currently recommended standards and underlying guidelines for rodent surgery—and finally to compile a comprehensive guideline of good surgical practice for rodent surgery.

**Search strategy:**

PubMed, Embase and Web of Science were searched to identify guidelines published in peer-reviewed journals. To identify grey literature and unpublished guidelines, we will perform a Google search for published guidelines and search laboratory animal sciences books for relevant book chapters. Additionally, we will conduct a survey among animal researchers enquiring about the guidelines they use.

**Screening and study selection:**

For publications retrieved by the systematic search, unique references are screened by two reviewers, first for eligibility based on title and abstract and subsequently for final inclusion based on full text. Eligibility of books is based on title and content, final inclusion based on chapter full text. Guidelines are either retrieved by Google searches or a survey. Google searches will be conducted by at least four of the authors. Thereafter, guidelines will be screened by two of the authors.

**Data extraction and synthesis:**

We will extract data from publications, book chapters and guidelines. Based on the extracted data, we will perform a descriptive synthesis of the bibliographical details, guideline development and endorsement, and the prevalence of individual recommendations, including subgroup analysis of the guidance per continent or country and differences between peer-reviewed versus non-peer-reviewed guidance.

**Strengths and limitations of this study**

- We use a systematic approach in order to obtain a more unbiased view on guidelines available for rodent surgery.
- We have defined inclusion and exclusion criteria in our protocol and blind the reviewers during data extraction.
- We try to review available information most comprehensively, not only including journal articles but also grey literature like book chapters and guidelines available in different formats.
- Our approach might be limited by the heterogeneity of the available information making it impossible to perform meta-analytical analyses on parameters.
- A number of internal guidelines might stay undetected because colleagues might not be able or allowed to share them.

## Introduction

Many experimental animal studies involve surgical procedures to induce a disease model, implant devices or to collect tissue or organ samples. Regardless of the surgical procedure, good surgical practice is the prerequisite for a successful outcome. Good surgical practice involves, but is not limited to (1) adequate surgical training prior to the planned surgery, (2) proper perioperative and postoperative care, (3) approved protocols of anaesthesia and analgesia, (4) approved surgical protocols and (5) the application of the principles of surgical asepsis. In its entirety, good surgical practice will result in safe, fast, minimally invasive and reproducible surgery, consequently minimising perioperative complications and improving post-surgical recovery as well as the validity of study outcomes.^1–3^

It is generally accepted in human as well as veterinary surgery, that aseptic technique used during surgery minimises the contamination with micro-organisms and thus prevents postoperative wound infection.^3 4^ The principles of good surgical practice were introduced nearly 200 years ago and—although methods, equipment and agents constantly were and still are refined throughout the years—surgical hand washing, sterile gowning and glowing as well as decontamination of the patient’s skin before surgery and the usage of sterile equipment is now standard practice in humans.^5 6^ However, protocols for surgical hygiene and good practice are not species-specific, and therefore, should be used regardless of performing surgery on human or animal patients of any size.

The legal requirements to perform experimental surgery are identical for rodents and large animals. For experimental rodent surgery, however, additional considerations must be taken into account, for example, large numbers of surgeries to be performed (‘batch surgery’), the use of genetically modified or immunocompromised rodents, the need for a specific (micro)surgical set up and dedicated instruments due to the animals’ small size. Additionally, there is often a very limited number of surgical assistants available.^7–10^

However, for rodents, the hygiene standards applied during surgery seem to be much lower, although it is known that rodents can develop (subclinical) wound infections and septicaemia—as they are used as infection models.^11–13^ Nevertheless, there seems to be a lack of implementation of good surgical practice during experimental rodent surgery. Therefore, the aim of this systematic review is to identify, critically evaluate and compare current guidelines describing good surgical practice for experimental rodent surgery. The results of this study will be used to compile a comprehensive guideline of good surgical practice for rodent surgery, which will then be promoted via laboratory animal organisations and societies as well as implemented into surgical training offers.

## Materials and methods

This protocol is reported according to the Preferred Reporting Items for Systematic Review and Meta-analysis-Protocol^14^ guidelines as far as possible (some elements, for example risk of bias assessment and meta-analysis, are not applicable to this systematic review of guidelines). At the time of submission of this protocol, full-text screening had been started, but not completed.

### Eligibility criteria

We aim to include all (types of) records describing guidelines on good surgical practice for rodents, regardless of language, publication status or date of publication. Languages included will be Dutch, English, French and German. Records not focusing on general surgical practice, but rather describing the details of a certain surgical intervention will not be considered. For both title and abstract and full-text screening, the following exclusion criteria are used: (1) not on animals (eg, human/in vitro); (2) not a guideline (eg, primary animal study); (3) not on surgery (eg, LAS guideline on another topic) and (4) on surgery, but not on rodents.

### Search strategies

#### Publications in peer-reviewed journals

**Figure 1:**
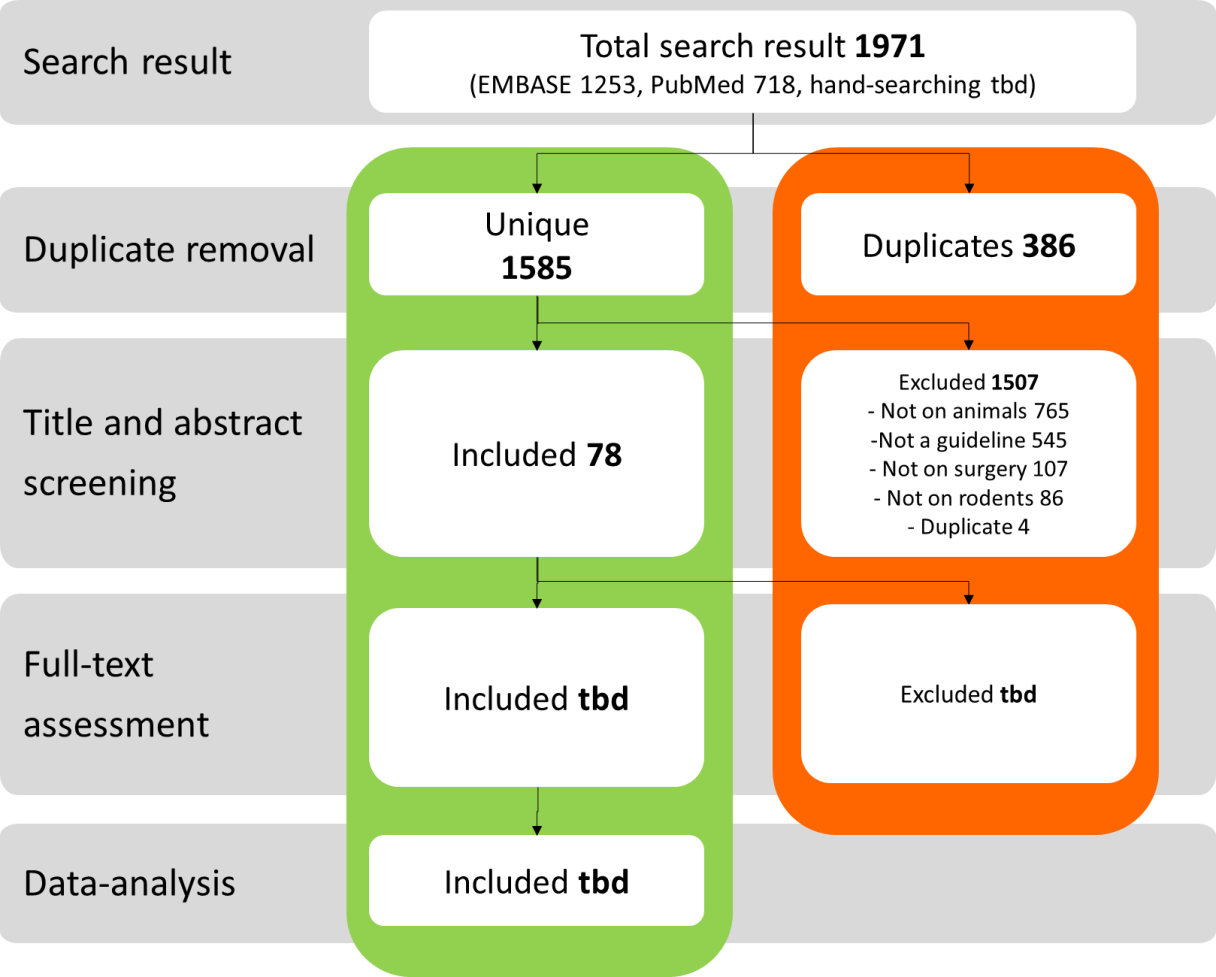
Search strategy and searches conducted so far for peer-reviewed journals.

PubMed and Embase (via Ovid) were searched from inception to 3 September 2021, to identify guidance on good surgical practice for rodent surgery published in peer-reviewed journals (figure 1). The comprehensive search strategy was based on the protocol published by Vollert *et al*.^15^ Search strings were based on the search components “guidelines”, “surgery” and “rodents”. The full comprehensive search strings are shown in tables 1 and 2. We will also manually search laboratory animal science handbooks used by our team and researchers in our network for additional potentially relevant book chapters containing guidance on rodent surgery.

**Table 1:** Comprehensive search strings EMBASE

EMBASE 03-09-2021	Total # hits: 1253
Rodent surgery (588 hits)	(animal surgery or rodent surgery or murine surgery or rat surgery or mouse surgery).ti, ab, kw. OR ((animal or animals or rodent or rodents or murine or rat or rats or mouse or mice) adj2 surgery).ti.
Guideline AND animal study (4812 hits)	(Consensus/ OR consensus development/ OR practice guideline/ OR position statement*.ti, ab, kw. OR policy statement*.ti, ab, kw. OR practice parameter*.ti, ab, kw. OR best practice*.ti, ab, kw. OR standards.ti. OR guideline.ti. OR guidelines.ti. OR recommendation.ti. OR recommendations.ti.) AND (Preclinical model OR Pre-clinical model OR Preclinical models OR Pre-clinical models OR disease model OR disease models OR animal model OR animal models OR experimental model OR experimental models OR preclinical study OR pre-clinical study OR preclinical studies OR pre-clinical studies OR animal study OR animal studies OR animal experiment* OR experimental study OR experimental studies).ti, ab, kw.)
Guideline AND (animal AND study) (2998 hits)	(Consensus/ OR consensus development/ OR practice guideline/ OR position statement*.ti, ab, kw. OR policy statement*.ti, ab, kw. OR practice parameter*.ti, ab, kw. OR best practice*.ti, ab, kw. OR standards.ti. OR guideline.ti. OR guidelines.ti. OR recommendation.ti. OR recommendations.ti.) AND ((preclinical.ti, ab, kw. OR Pre-clinical.ti, ab, kw. OR experimental.ti, ab, kw. OR animal.ti, ab, kw.) adj2 (study.ti, ab, kw. OR studies.ti, ab, kw. OR model.ti, ab, kw. OR models.ti, ab, kw.)) AND (rodent OR rodents OR rat OR rats OR mouse OR mice OR murine OR animal).mp.
Methods AND tool AND (animal AND study) (248 hits)	(methodology/ OR experimental design/ OR study design/) AND (tool.ti. OR protocol.ti.) AND (exp animal experiment/ OR exp animal model/ OR ((preclinical.ti, ab, kw. OR pre-clinical.ti, ab, kw. OR experimental.ti, ab, kw. OR animal.ti, ab, kw.) adj2 (study.ti, ab, kw. OR studies.ti, ab, kw. OR model.ti, ab, kw. OR models.ti, ab, kw.))) AND (rodent OR rodents OR rat OR rats OR mouse OR mice OR murine OR animal).mp.
Surgery (6 685 256 hits)	exp surgery/ OR exp veterinary surgery/ OR exp experimental surgery/ OR (surgery OR surgeries OR surgical OR operation OR operations OR operative OR reoperation OR re-operation OR aseptic technique OR aseptic techniques OR Abdominoplasty OR Acetabuloplasty OR Adenoidectomy OR Adrenalectomy OR Amputation OR Anastomosis OR Apicoectomy OR Appendectomy OR Arthrodesis OR Arthroplasty OR Biopsy OR Blepharoplasty OR Bypass OR Castration OR Cementoplasty OR Cervicoplasty OR Cholecystostomy OR Choledochostomy OR Colectomy OR Colpotomy OR Craniectomy OR Craniotomy OR Curettage OR Cystostomy OR Cytoreduction OR Dacryocystorhinostomy OR Debridement OR Denervation OR Diskectomy OR Discectomy OR Dissection OR Electrosurgery OR Enterostomy OR Esophagectomy OR Esophagoplasty OR Esophagostomy OR Excision OR Fasciotomy OR Foraminotomy OR Fundoplication OR Gastrectomy OR Gastroenterostomy OR Gastropexy OR Gastroplasty OR Gastrostomy OR Gingivectomy OR Gingivoplasty OR Glossectomy OR Grafting OR Hemorrhoidectomy OR Hepatectomy OR Herniorrhaphy OR Hypophysectomy OR Hysterotomy OR Iridectomy OR Keratectomy OR Laminectomy OR Laminoplasty OR Laparotomy OR Laryngectomy OR Laryngoplasty OR Lipectomy OR Lobectomy OR Mammaplasty OR Mastectomy OR Mediastinoscopy OR Meniscectomy OR Metastasectomy OR Microdissection OR Microsurgery OR Myotomy OR Nanosurgery OR Nephrostomy OR Neurosurgery OR Osteotomy OR Ostomy OR Pallidotomy OR Pancreatectomy OR Pancreaticoduodenectomy OR Pancreaticojejunostomy OR Parathyroidectomy OR Pharyngectomy OR Pinealectomy OR Pneumonectomy OR Portoenterostomy OR Proctectomy OR Psychosurgery OR Puncture OR Pyloromyotomy OR Resection OR Replantation OR Rhinoplasty OR Salpingostomy OR Scleroplasty OR Sclerostomy OR Shunt OR Sphincterotomy OR Splenectomy OR Sterilization OR Sternotomy OR Symphysiotomy OR Synovectomy OR Tenodesis OR Tenotomy OR Thoracoplasty OR Thoracostomy OR Thoracotomy OR Thymectomy OR Thyroidectomy OR Tonsillectomy OR Tracheostomy OR Tracheostomy OR Tracheotomy OR Transplantation OR Ureterostomy OR Vasovasostomy OR Vitrectomy).ti, ab, kw.
1 OR 2 OR 3 OR 4	5856
5 AND 6	1802
Limit to relevant publication types (1253 hits)	limit 6 to (article or article in press or books or chapter or conference paper or “conference review” or erratum or “review”)

**Table 2:** Comprehensive search strings PubMed

Pubmed 03-09-2021	Total # hits: 718
Rodent surgery (239 hits)	“animal surgery”[tiab] or “rodent surgery”[tiab] or “murine surgery”[tiab] or “rat surgery”[tiab] or “mouse surgery”[tiab] OR “surgery in animals”[tiab] OR “surgery in rodents”[tiab] OR “surgery in rats”[tiab] OR “surgery in mice”[tiab] OR “animal survival surgery”[tiab] OR “rodent survival surgery”[tiab] OR “rat survival surgery”[tiab] OR “mouse survival surgery”[tiab]
Guideline AND animal study (1719 hits)	(“Consensus”[Mesh] OR “Consensus development conferences as topic”[Mesh] OR “Guidelines as topic”[Mesh] OR “Practice guidelines as topic”[Mesh] OR guideline[pt] OR practice guideline[pt] OR consensus development conference[pt] OR position statement*[tiab] OR policy statement*[tiab] OR practice parameter*[tiab] OR best practice*[tiab] OR standards[ti] OR guideline[ti] OR guidelines[ti] OR recommendation[ti] OR recommendations[ti] OR “practical guideline”[tiab]) AND (Preclinical model[tiab] OR Pre-clinical model[tiab] OR Preclinical models[tiab] OR Pre-clinical models[tiab] OR disease model[tiab] OR disease models[tiab] OR animal model[tiab] OR animal models[tiab] OR experimental model[tiab] OR experimental models[tiab] OR preclinical study[tiab] OR pre-clinical study[tiab] OR preclinical studies[tiab] OR pre-clinical studies[tiab] OR animal study[tiab] OR animal studies[tiab] OR animal experiment*[tiab] OR experimental study[tiab] OR experimental studies[tiab])
Guideline AND (animal AND study AND animal mesh) (1983 hits)	(“Consensus”[Mesh] OR “Consensus development conferences as topic”[Mesh] OR “Guidelines as topic”[Mesh] OR “Practice guidelines as topic”[Mesh] OR guideline[pt] OR practice guideline[pt] OR consensus development conference[pt] OR position statement*[tiab] OR policy statement*[tiab] OR practice parameter*[tiab] OR best practice*[tiab] OR standards[ti] OR guideline[ti] OR guidelines[ti] OR recommendation[ti] OR recommendations[ti] OR “practical guideline”[tiab]) AND ((preclinical[tiab] OR pre-clinical[tiab] OR experimental[tiab] OR animal[tiab] OR rodent[tiab] OR rodents[tiab] OR rat[tiab] OR rats[tiab] OR mouse[tiab] OR mice[tiab] OR murine[tiab]) AND (study[tiab] OR studies[tiab] OR model[tiab] OR models[tiab]) AND (“Animals”(Mesh:noexp)OR “Mice”[Mesh] OR “Rats”[Mesh] OR “Rodentia”[Mesh]))
Methods AND tool AND (animal AND study) (1660 hits)	((“Methods”[Mesh] OR “Methods”(Subheading)) AND (tool[ti] OR protocol[ti])) AND (“Animal Experimentation”[Mesh] OR “Models, Animal”[Mesh] OR ((preclinical[tiab] OR pre-clinical[tiab] OR experimental[tiab] OR animal[tiab] OR rodent[tiab] OR rodents[tiab] OR rat[tiab] OR rats[tiab] OR mouse[tiab] OR mice[tiab] OR murine[tiab]) AND (study[tiab] OR studies[tiab] OR model[tiab] OR models[tiab])) AND (“Animals”[Mesh:noexp]OR “Mice”[Mesh] OR “Rats”[Mesh] OR “Rodentia”[Mesh]))
Guideline AND (animal AND study NOT medline) (584 hits)	(position statement*[tiab] OR policy statement*[tiab] OR practice parameter*[tiab] OR best practice*[tiab] OR standards[ti] OR guideline[ti] OR guidelines[ti] OR recommendation[ti] OR recommendations[ti] OR “practical guideline”[tiab]) AND ((preclinical[tiab] OR pre-clinical[tiab] OR experimental[tiab] OR animal[tiab] OR rodent[tiab] OR rodents[tiab] OR rat[tiab] OR rats[tiab] OR mouse[tiab] OR mice[tiab] OR murine[tiab]) AND (study[tiab] OR studies[tiab] OR model[tiab] OR models[tiab])) NOT medline[sb]
Surgery (5 101 762 hits)	“Surgical Procedures, Operative”[Mesh] OR “General Surgery”[Mesh] OR “Surgery, Veterinary”[Mesh] OR surgery[tiab] OR surgeries[tiab] OR surgical[tiab] OR operation[tiab] OR operations[tiab] OR operative[tiab] OR reoperation[tiab] OR re-operation[tiab] OR aseptic technique[tiab] OR aseptic techniques[tiab] OR Abdominoplasty[tiab] OR Acetabuloplasty[tiab] OR Adenoidectomy[tiab] OR Adrenalectomy[tiab] OR Amputation[tiab] OR Anastomosis[tiab] OR Apicoectomy[tiab] OR Appendectomy[tiab] OR Arthrodesis[tiab] OR Arthroplasty[tiab] OR Biopsy[tiab] OR Blepharoplasty[tiab] OR Bypass[tiab] OR Castration[tiab] OR Cementoplasty[tiab] OR Cervicoplasty[tiab] OR Cholecystostomy[tiab] OR Choledochostomy[tiab] OR Colectomy[tiab] OR Colpotomy[tiab] OR Craniectomy[tiab] OR Craniotomy[tiab] OR Curettage[tiab] OR Cystostomy[tiab] OR Cytoreduction[tiab] OR Dacryocystorhinostomy[tiab] OR Debridement[tiab] OR Denervation[tiab] OR Diskectomy[tiab] OR Discectomy[tiab] OR Dissection[tiab] OR Electrosurgery[tiab] OR Enterostomy[tiab] OR Esophagectomy[tiab] OR Esophagoplasty[tiab] OR Esophagostomy[tiab] OR Excision[tiab] OR Fasciotomy[tiab] OR Foraminotomy[tiab] OR Fundoplication[tiab] OR Gastrectomy[tiab] OR Gastroenterostomy[tiab] OR Gastropexy[tiab] OR Gastroplasty[tiab] OR Gastrostomy[tiab] OR Gingivectomy[tiab] OR Gingivoplasty[tiab] OR Glossectomy[tiab] OR Grafting[tiab] OR Hemorrhoidectomy[tiab] OR Hepatectomy[tiab] OR Herniorrhaphy[tiab] OR Hypophysectomy[tiab] OR Hysterotomy[tiab] OR Iridectomy[tiab] OR Keratectomy[tiab] OR Laminectomy[tiab] OR Laminoplasty[tiab] OR Laparotomy[tiab] OR Laryngectomy[tiab] OR Laryngoplasty[tiab] OR Lipectomy[tiab] OR Lobectomy[tiab] OR Mammaplasty[tiab] OR Mastectomy[tiab] OR Mediastinoscopy[tiab] OR Meniscectomy[tiab] OR Metastasectomy[tiab] OR Microdissection[tiab] OR Microsurgery[tiab] OR Myotomy[tiab] OR Nanosurgery[tiab] OR Nephrostomy[tiab] OR Neurosurgery[tiab] OR Osteotomy[tiab] OR Ostomy[tiab] OR Pallidotomy[tiab] OR Pancreatectomy[tiab] OR Pancreaticoduodenectomy[tiab] OR Pancreaticojejunostomy[tiab] OR Parathyroidectomy[tiab] OR Pharyngectomy[tiab] OR Pinealectomy[tiab] OR Pneumonectomy[tiab] OR Portoenterostomy[tiab] OR Proctectomy[tiab] OR Psychosurgery[tiab] OR Puncture[tiab] OR Pyloromyotomy[tiab] OR Replantation[tiab] OR Rhinoplasty[tiab] OR Resection[tiab] OR Salpingostomy[tiab] OR Scleroplasty[tiab] OR Sclerostomy[tiab] OR Shunt[tiab] OR Sphincterotomy[tiab] OR Splenectomy[tiab] OR Sterilisation[tiab] OR Sternotomy[tiab] OR Symphysiotomy[tiab] OR Synovectomy[tiab] OR Tenodesis[tiab] OR Tenotomy[tiab] OR Thoracoplasty[tiab] OR Thoracostomy[tiab] OR Thoracotomy[tiab] OR Thymectomy[tiab] OR Thyroidectomy[tiab] OR Tonsillectomy[tiab] OR Tracheostomy[tiab] OR Tracheostomy[tiab] OR Tracheotomy[tiab] OR Transplantation[tiab] OR Ureterostomy[tiab] OR Vasovasostomy[tiab] OR Vitrectomy[tiab]
(1 OR 2 OR 3 OR 4) AND 5	718

#### Grey literature (book chapters and unpublished guidelines)

We will search laboratory animal sciences books for relevant book chapters. Books will be included based on their title and content, the relevant chapters will be screened by two of the authors, the final inclusion will be based on the chapter full-text. In addition to published guidelines, we anticipate that researchers may also use unpublished (institutional) guidelines or protocols for rodent surgery. We will therefore supplement our systematic search of published literature with two strategies to identify grey literature. First, we will perform a Google search to identify (institutional) guidelines posted on, for example, university websites. We will use various combinations and variations on the following search terms: rodent, mouse, rat, surgery, aseptic, guidance, guidelines and protocol (in Dutch, English, French and German) and will screen at least the first 50 search results for each search performed. Google searches will be performed by at least four authors with at least four different computers to take into account that search engines customise to computers. Second, we will attempt to obtain local institutional guidelines by approaching animal researchers in our extended network or through learnt societies such as the Federation of European Laboratory Animal Science Associations, the European College for Laboratory Animal Medicine, the European Society of Laboratory Animal Veterinarians, Norway’s National Consensus Platform for the advancement of the 3 Rs (Norecopa), NC3R and Understanding Animal Research with a request to (anonymously) submit guidelines for rodent surgery.

### Screening and study selection

For publications, duplicates were removed from the combined comprehensive search results using ASYSD (https://camarades.shinyapps.io/RDedup/). Screening of unique records is performed using the Rayyan platform (https://rayyan.qcri.org/). In both screening phases, each record is screened independently by two reviewers (PS and either KEW, FG or SZ), who are blinded to each other’s decisions, but not to the authors of the records being screened. Discrepancies will be resolved through discussion, or, if no consensus can be reached, a third reviewer will serve as arbiter.

We will attempt to obtain full-text versions of all included articles through open access, interlibrary loan or by contacting authors directly. Articles for which no full-text version can be obtained will be excluded from the review. We will check the reference lists of included studies and book chapters and relevant reviews for additional eligible references based on title, which will then undergo screening as described above.

For grey literature, potentially relevant book chapters and documents obtained by Google search as well as submitted by survey respondents will be included based on their title, content and structure of the relevant chapters by the reviewer retrieving them. Details on the source, title, author or institute of the records will be recorded in a spreadsheet and duplicates will be detected manually. Eligible records will then be assessed by a second reviewer for final inclusion. All included records will be then distributed to two independent reviewers and screened based on full-text using the same exclusion criteria as described above.

### Data extraction and synthesis

From each included record (ie, publications, book chapters or guidelines), we will extract bibliographical details, for example, first author, country of institute of the first author and year of publication and journal. From each record, we will then extract all its individual guidance elements as individual recommendations. Based on the experience of the review team, a preliminary list of individual recommendations has been created (see tables 3 and 4), and we will extract data on whether or not guidelines contain these recommendations. Second, we will record any additional recommendations not yet included in the list. Across guidelines, the elements will be ranked based on the frequency of appearance across the included guidelines. Finally, we will extract characteristics pertaining to the development of and support for the record, to come to a diligence classification (table 5).

**Table 3:** Data extraction form for individual recommendations per record—part 1

Guideline ID: (author_year)or(institute_city_country) Guideline title: Reviewed by:				
**#. Topic**	**Sub-topic (Reported, Y/N**)		**Quote from guideline**	**References to primary research**
1. Facility/room	□ No specific recommendation			
	□ Dedicated operating area/room			
	□ Separate preparation area/room			
	□ other ________			
2. Equipment	□ Magnification (Microscope, Magnifying glasses?)			
	□ Dedicated instruments			
	□ Warming equipment			
	□ Monitoring equipment			
	□ other ________			
3. Type of surgery	□ survival	□ superficial (s.c.)		
	□ non-survival	□ minor		
		□ major		
		□ stereotaxic		
4. Duration of surgery	□ < 30 min	□ 1–2 hours		
	□ 30 min to 1 hour	□ > 2 hours		
	□ not specified			
5. Sterile Instruments	□ yes			
	□ no/not mentioned			
6. Sterile consumables	□ yes			
	□ not mentioned			
7. Instrument storage	□ Not mentioned			
	□ Instrument case, pouches			
8. Instrument rest area	□ Wiping/disinfection			
	□ Draping			
	□ Sterile draping			
	□ other ________			
9. Batch surgery	□ Not mentioned			
	□ New set of sterile instruments/animal			
	□ Glass bead steriliser between animals			
	□ Wiping with alcohol in between animals			
	□ other ________			
10. Clothing	□ Bonnet			
	□ Face mask			
	□ Sterile surgical gown			
	□ Sterile surgical gloves			
	□ other ________			
11. Surgeon’s routine	□ Hand washing			
	□ Hand disinfection			
	□ Sterile gowning			
	□ Sterile gloving			
	□ other ________			

**Table 4:** Data extraction form for individual recommendations per record—part 2

Guideline ID: (author_year) or (institute_city_country)				
Guideline title:				
Reviewed by:				
**#. Topic**	**Sub-topic (Reported, Y/N**)		**Quote from guideline**	**References to primary research**
12. Animal:	□ Not mentioned/no removal			
Fur removal	□ Electric clipper			
	□ Wet shave (razor / blade)			
	□ Depilation cream			
	□ other ________			
13. Surgical field	□ Washing (soap)			
	□ Disinfection with alcohol			
	□ Combined wiping/disinfection			
	□ other ________			
14. Draping	□ Draping			
	□ Sterile draping			
	□ Cling foil			
	□ other ________			
15. Wound closure	□ Threads			
	□ Clips			
	□ Glue			
	□ other ________			
16. Wound care	□ Not mentioned			
	□ (Regular) Cleaning			
	□ (Regular) Disinfection			
	□ Application of ointment			
17. Antibiotics	□ Not mentioned			
	□ Mentioned: What, When, How long?			
18. Analgesics	□ single	□ local/topical		
		□ NSAIDs		
	□ multimodal	□ Opiods		
		□ Others (what?)		
19. Assistance recommended	□ For preparation			
	□ During surgery			
	□ For anaesthesia/monitoring			
	□ For postoperative care			
20. Training recommended	□ For aseptic technique			
	□ For surgical procedure			
	□ For anaesthesia			
	□ other ________			
21. Time published	□ prior to 1991			
	□ 1991–2000			
	□ 2001–2010			
	□ 2011–2020			
	□ 2021-			

**Table 5:** Data extraction form for diligence classification of guidelines

Guideline ID	Evidence-based development process			Support	
	Recommendations based on a systematic review (Y/N)	Recommendation by groups of individuals, through a method which included a Delphi process or other means of structured decision-making (Y/N)	Recommendations of individuals or small groups of individuals based on individual experience only (Y/N)	Published stand alone (Y/N)	Endorsed or initiated by at least one publisher or scientific society (Y/N)
(author_year)					
(author_year)					
Etc					

All data will be extracted by one reviewer and checked for errors by a second reviewer. In case of discrepancies, the initial two reviewers will attempt to reach consensus through discussion. If consensus cannot be reached, a third reviewer will serve as arbiter. Subsequently, we will perform a descriptive synthesis of the bibliographical details, characteristics of guideline development and endorsement, and the prevalence of (themes of) individual recommendations. We will perform subgroup analysis of the guidance per continent, within Europe per country and differences between peer-reviewed versus non-peer-reviewed guidance.
